# Performance of Integrated Emergency Surgical Officers at Mizan-Tepi University Teaching Hospital, Mizan-Aman, Ethiopia: A Retrospective Cohort Study

**DOI:** 10.1155/2021/8875560

**Published:** 2021-01-05

**Authors:** Margo S. Harrison, Ephrem Kirub, Tewodros Liyew, Biruk Teshome, Andrea Jimenez-Zambrano, Margaret Muldrow, Teklemariam Yarinbab

**Affiliations:** ^1^University of Colorado School of Medicine, Aurora, Colorado, USA; ^2^Mizan-Tepi University Department of Public Health, Mizan-Aman, Ethiopia; ^3^Village Health Partnership, Denver, Colorado, USA

## Abstract

**Introduction:**

Surgical physician extenders are used in Ethiopia and sub-Saharan Africa where there is a lack of surgical providers.

**Methods:**

We tested characteristics associated with and outcomes of births attended by an integrated emergency surgical officers (IESOs) as compared to midwives and physician providers.

**Results:**

Of 1,000 women in our convenience sample, data on birth attendant was missing on 5 women (0.5%). Of the remaining women, almost three-fourths (73.6%, *n* = 732) of women were attended by a midwife, almost a quarter were attended by an IESO (24.4%, *n* = 243), 10 women were attended by a physician with a General Practitioner level of training (1.0%), 5 women were delivered by an Ob/Gyn resident (0.5%), and 5 women were attended by an Ob/Gyn (0.5%). Women had a higher likelihood of being attended by an IESO than a midwife if they underwent forceps-assisted (RR 88.4, *p* < 0.05), vacuum-assisted (RR 45.2, *p* < 0.05), or cesarean birth (RR 161.8, *p* < 0.05) as compared to an unassisted vaginal birth. IESOs are performing more operative vaginal and cesarean births than other delivery providers. Outcomes of their deliveries are worse than those of midwives, but this is likely due to the acuity level of the patients and not the provider type.

## 1. Introduction

While cesarean birth rates are increasing globally, many regions still experience unacceptably low cesarean birth rates [1, 2]. Overuse and underuse of cesarean birth can often coexist within countries and even communities [3, 4]. Though many urban areas in sub-Saharan Africa may report acceptable cesarean birth rates, many regions remain at risk for low cesarean birth rates [5–7]. One issue cited as a contributing factor to low cesarean birth rates in sub-Saharan Africa is lack of a sufficiently trained and available surgical work force [8]. One innovative solution to this crisis has been surgical task-shifting, or the training of nonphysician providers to deliver emergency surgery, including cesarean birth [9–11]. In Ethiopia, a cadre of midlevel providers referred to as Integrated Emergency Surgical Officers (IESOs) has been trained to fill the gap in availability of surgical providers [12].

Ethiopia has already increased surgical staff nationally through task-shifting emergency surgery to IESOs [9, 10, 12–15]. The program was introduced in 2009; IESOs pursue a three-year course in emergency obstetrics and general surgery. [12] From 2012–2014, 4,075 operations were performed by IESOs, 63% of which were cesarean births. During this timeframe the cesarean birth rate was 12.5% (within the WHO recommended 10–15% range) [12, 16–18]. Qualitative results reported that staff “stressed that maternal and child health improved significantly after the IESOs were assigned” and that IESOs “made emergency surgery services accessible to the majority, and their clinical decision-making and surgical skills were remarkable” [12]. Minimal literature exists on the performance of IESOs beyond this initial evaluation [12]. As such, we wished to observe the contribution and performance of IESOs at Mizan-Tepi University Teaching Hospital as a contribution to the literature.

## 2. Methodology

We conducted a hospital-based, prospective cross-sectional study at Mizan-Tepi University Teaching Hospital (MTUTH), which is located in Mizan-Aman in the Southern Nations, Nationalities, and People's Region (SNNPR), Ethiopia [19]. The study population was a convenience sample of all women who delivered at MTUTH between May 6 and October 21, 2019, which was the point at which 1,000 women were included in the cohort [19]. Only mothers who delivered after 28 weeks gestational age were included [19]. Deidentified data was collected by highly trained physicians with the objective of planning future quality improvement and research interventions [19]. Physicians were involved in study design, but patients were not [19]. A combination of structured interview and chart review was used to collect patient information upon admission, delivery, and discharge [19]. Data was collected on paper forms, which were reviewed for completeness prior to data entry into REDCap [19]. Data was then electronically transmitted for secure storage on a password protected server at the University of Colorado, Aurora, Colorado, USA [19, 20].

STATA software version 15.2 (StataCorp LP, College Station, TX, USA) was used for analysis [19]. Bivariate comparisons of sociodemographic, obstetric, labor, delivery, and pregnancy outcomes of women delivered by midwives versus IESOs and women delivered by IESOs versus physician providers were performed [19]. We utilized Fisher's exact, Chi-squared, and Kruskal–Wallis tests depending on the variables [19]. All covariates significant to *p* < 0.05 in bivariate comparisons were included in a multivariable Poisson model with robust error variance (because IESO attendance was prevalent) to determine which covariates were independently associated with IESO delivery [19]. Subsequently, individual logistic regressions (because the outcomes were not as prevalent) of maternal and perinatal outcomes (significant in the bivariate comparisons) were run with the outcomes as the dependent variable and IESO attendant as the independent variable, adjusted for all covariates significant in the multivariable Poisson model, to describe the association between IESO attendance and adverse pregnancy outcomes [19].

This quality improvement survey was given an exempt from human subjects' research approval (COMIRB # 18-2738) by the University of Colorado and approval [19]. Despite the quality improvement nature of the work and the fact that only deidentified data was collected, oral consent was obtained from each woman before any of her data was recorded [19].

## 3. Results

**Figure 1:**
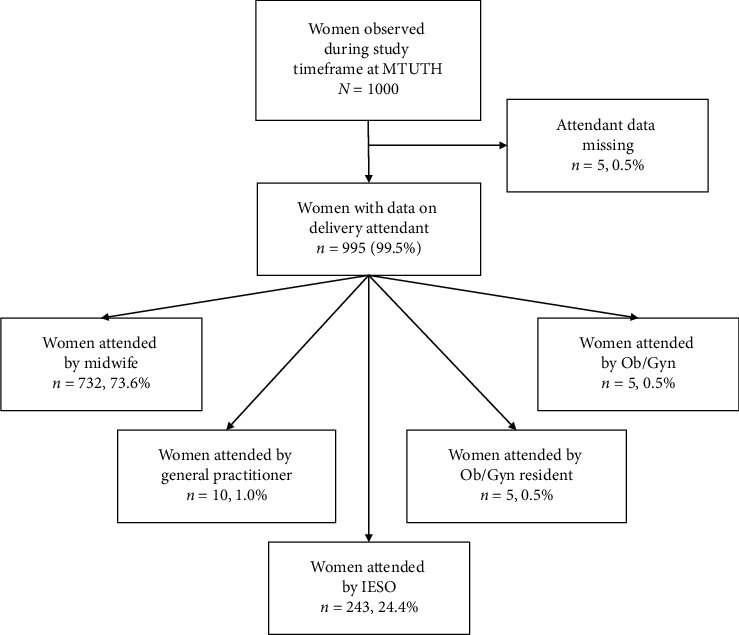
Study population by attendant.

As shown in Figure 1, of 1,000 women on whom data was collected, 995 included information on delivery attendant (who delivered the mother). Almost three-fourths (73.6%, *n* = 732) of women were attended by a midwife, almost a quarter were attended by an IESO (24.4%, *n* = 243), 10 women were attended by a physician with a General Practitioner level of training (1.0%), 5 women delivered by an Ob/Gyn resident (0.5%), and 5 women were attended by an Ob/Gyn (0.5%).

Table 1 initially describes the overall population of women delivered by a midwife or an IESO and then compares the two groups by these provider types. The median age of women was 24 years (interquartile range [IQR] 20, 28), the largest subgroup (39.6%) has a primary school level of education, a majority of women were of Protestant religion (54.3%), 96.4% were not single, over half (54.0%) lived in an urban setting, many were nulliparous (43.4%), the majority had an interpregnancy interval of at least 24 months (52.1%), human immunodeficiency virus (HIV) was not very prevalent (2.1%), and median number of prenatal visits was 4 (IQR 3, 5). In bivariate comparisons of women who delivered by a midwife versus an IESO, women who delivered by an IESO were more likely to live in an urban area, be nulliparous, and have a history of cesarean birth; they were less likely to have HIV, *p* < 0.05.

The table then goes on to show these same comparison groups but with reference to antepartum, labor, and delivery characteristics. Overall the majority of group went into spontaneous labor (85.2%), were not transferred during labor (50.9%), were in labor less than 12 hours (51.5%), and did not experience antepartum hemorrhage (97.7%), chorioamnionitis (99.4%), or hypertensive diseases of pregnancy (95.3%). The largest subgroup was admitted in latent labor (49.8%) as compared to active, which is defined in this setting as 4 centimeters (47.2%), and the majority of births were unassisted vaginal deliveries (72.6%). Most women were term at birth (89.2%) and most babies weighed at least 2500 grams (88.0%) and were singleton gestations (95.2%). In bivariate comparisons, women who delivered by an IESO were more likely to have their labor be augmented and induced, or labor was “not applicable” (suggesting cesarean birth); they were more likely to have been transferred to MTUTH, to have a labor over 12 hours in duration, to experience antepartum hemorrhage, to undergo cesarean birth, to have a larger median size of their infant in grams, and to deliver singleton gestations, *p* < 0.05.

In the final section of Table 1, postpartum complications of the overall cohort as well as the comparison groups are presented. Though more complications were tested (see table notes), all adverse outcomes shown to be statistically significantly different by attendant time are tabulated. Women who delivered by an IESO had higher rates of maternal blood transfusion, a higher need for postpartum antibiotics, and a longer maternal hospitalization, *p* < 0.05. Regarding neonatal outcomes, delivery by an IESO resulted in higher rates of fresh stillbirth, higher rates of neonatal demise, and a longer neonatal hospitalization, *p* < 0.05.

Table 2 shows the results of our multivariable modeling. Of all significant antepartum covariates, only mode of delivery was associated with delivery attendant. Women had a higher relative risk of being attended by an IESO if they underwent forceps-assisted (RR 88.4, *p* < 0.05), vacuum-assisted (RR 45.2, *p* < 0.05), or cesarean birth (RR 161.8, *p* < 0.05) as compared to an unassisted vaginal birth. When individual logistic regressions of all postpartum complications significant in Table 1 adjusted for mode of delivery were performed, odds of need maternal postpartum antibiotics (OR 13.4, *p* < 0.05), odds of having a lower neonatal Apgar score (OR 0.1, *p* < 0.05), and odds having a live neonate on discharge from the hospital (OR 0.1, *p* < 0.05) were associated with IESO delivery as compared to a midwife attendant.

Table 3 is formatted the same way as Table 1 but compares deliveries by IESOs to those performed by a physician provider (General Practitioner [GP] or Ob/Gyn (MD)). In bivariate comparisons, the groups were not different by sociodemographic or obstetrical history characteristics although there was borderline difference in religion with a higher proportion of women delivered by physicians identifying as Protestant (*p*=0.05). In the second section, the groups were also borderline different in terms of the onset of labor of patients with patients delivered by IESOs more likely to be spontaneous or augmented/induced compared to “not applicable,” which likely represents cesarean birth (*p*=0.05). The groups were different on antepartum hemorrhage with more women who delivered by IESOs experiencing this complication and by mode of delivery with more women who delivered by IESO experiencing cesarean birth, *p* < 0.05. Though many postpartum complications were tested (see table notes) only the lengths of maternal and neonatal hospitalization were different between groups with women who delivered by IESOs having a longer hospitalization, *p* < 0.05.

Table 4 shows the results of multivariable modeling. As compared to Muslim women, Catholic women were less likely to deliver by an IESO as compared to a physician (RR 0.9, *p* < 0.05). Compared to women whose onset of labor was “not applicable,” those in spontaneous labor (RR 1.3, *p* < 0.05) were more likely to deliver by an IESO. With respect to mode of delivery, women with vacuum-assisted (RR 2.4, *p* < 0.05) or cesarean birth (RR 2.3, *p* < 0.05) were more likely to have an IESO attendant than a physician attendant. In a logistic regression adjusted for religion, labor onset, and mode of delivery, being delivered by an IESO was not associated with length of neonatal hospitalization as compared to delivery by a physician provider.

## 4. Discussion

Most women at MTUTH are delivered by a midwife, but those requiring a higher level of care are majority attended to by IESOs, with very few women in our cohort delivered by physicians. Compared to midwives, IESOs are experiencing a higher need for postpartum antibiotics and are more likely to have neonates with lower median Apgars, who are more likely to demise before discharge from the hospital. As compared to physician birth attendants, IESOs did not experience any more postpartum complications and interestingly were more likely to provide cesarean and vacuum delivery than physicians.

The fact that IESOs delivered women with complications such as transfer during the labor course, prolonged labor, and antepartum hemorrhage suggests that they are functioning as a higher acuity labor and delivery provider than midwives, which is consistent with their training. It also implies that appropriate risk stratification is happening on the labor and delivery service at MTUTH and that when women experience complications, their care is being escalated. We find this reassuring and consistent with prior research findings [12]. Further observational research would be interesting to observe this transition of care and whether it is protocol-based or determined mostly by clinical decision-making as this would be an important process to disseminate to settings training and leveraging midlevel providers to extend surgical care.

Outcomes, including severe adverse neonatal outcomes like neonatal demise, were worse among IESOs as compared to midwives. Likely, as per above, this has to do with IESOs taking care of sicker patients who are experiencing antepartum complications and are more likely to require operative or surgical delivery, which may result in adverse neonatal outcomes. As this analysis is limited by the covariates collected, secondary analysis is unlikely to give a full understanding of what accounts for this finding. This would be an area where quality improvement work could likely significantly contribute to understanding this outcome and provide additional information on how to improve neonatal outcomes related to labor and delivery care and what risk is attributable to the delivery provider versus other factors.

In our analysis of IESOs versus physician providers, there were minimal clinically significant characteristics in the patient populations served by these providers (IESOs did seem to take care of more complicated patients) and no discernable difference in pregnancy outcomes. This finding may be due to the fact that the IESOs are providing a high-quality level of care or may be explained by the fact that very few deliveries were performed by physicians and as such there was not sufficient sample size to determine any differences. It was curious that there was a difference in the populations based on religion; this may be a spurious association, but it would be worth further quality improvement review to ensure that care in the facility is not variable by religious affiliation of the woman.

This analysis was limited in that we could only consider the variables that were assessed in our dataset, and the dataset was designed to look at many pregnancy outcomes, not just delivery attendant. There is more that needs to be understood about poor neonatal outcomes related to births by IESOs that cannot be determined with this dataset and requires further evaluation. It should be noted that the poor neonatal outcomes may be explained by other factors other than delivery attendant.

In summary, IESOs appear to be extending care at MTUTH beyond a midwifery level of care, and their care is associated with good maternal and neonatal outcomes. Work on improving the postpartum infection rate in the facility and better understanding adverse neonatal outcomes are areas requiring further research. Additional observational data on the role of the IESO in the labor course would also be of interest for dissemination purposes.

## Figures and Tables

**Table 1:** Sociodemographic and obstetric characteristics of women overall and by midwife versus IESO attendant.

Characteristic	*N* (%) *N* = 975	Midwife (*n* = 732, 75.1%)	IESO (*n* = 243, 24.9%)	*p* value
Sociodemographic & obstetric history characteristics				
Age in years, median (IQR)	24 [20, 28]	24 [20, 28]	25 [21, 28]	0.12^a^
Missing	1 (%)	1	0	

Education				0.54^c^
Unable to read & write	229 (23.5%)	165 (22.5%)	64 (26.3%)	
Read & write only	52 (5.3%)	44 (6.0%)	8 (3.3%)	
Primary school	386 (39.6%)	290 (39.6%)	96 (39.5%)	
Secondary school	138 (14.2%)	105 (14.4%)	33 (13.6%)	
Higher education	169 (17.3%)	127 (17.4%)	42 (17.3%)	
Missing	1 (0.1%)	1 (0.1%)	0 (0.0%)	

Religion				0.58^b^
Muslim	111 (11.4%)	77 (10.4%)	34 (14.0%)	
Orthodox christian	331 (34.0%)	252 (34.4%)	79 (32.5%)	
Catholic christian	0 (0.0%)	0 (0.0%)	0 (0.0%)	
Protestant	530 (54.3%)	400 (54.6%)	130 (53.5%)	
Jehovah's witness	2 (0.2%)	2 (0.3%)	0 (0.0%)	
Missing	1 (0.1%)	1 (0.1%)	0 (0.0%)	

Relationship status				0.67^b^
Single	27 (2.8%)	22 (3.0%)	5 (2.1%)	
Not single	940 (96.4%)	703 (96.0%)	237 (97.5%)	
Missing	8 (0.8%)	7 (1.0%)	1 (0.4%)	

Woreda				<0.001^c^
Urban	527 (54.0%)	368 (50.3%)	159 (65.4%)	
Rural	448 (46.0%)	364 (49.7%)	84 (34.6%)	

Parity				0.03^c^
0	423 (43.4%)	312 (42.6%)	111 (45.7%)	
1	253 (26.0%)	191 (26.1%)	62 (25.5%)	
2	140 (14.3%)	118 (16.1%)	22 (9.0%)	
3+	158 (16.2%)	110 (15.1%)	48 (19.8%)	
Missing	1 (0.1%)	1 (0.1%)	0 (0.0%)	

Months since last delivery (parity 1+ *n* = 552)				0.37^c^ 0.99^a^
<24 months	44 (4.5%)	36 (4.9%)	8 (3.3%)	
24+ months	508 (52.1%)	385 (52.6%)	123 (50.6%)	
Missing	423 (43.4%)	311 (42.5%)	112 (46.1%)	
Median (IQR)	60 [36, 84]	60 [36, 84]	48 [36, 84]	

History of cesarean birth				<0.001^b^
0	930 (95.4%)	716 (97.8%)	214 (88.1%)	
1	38 (3.9%)	13 (1.8%)	25 (10.3%)	
2+	5 (0.5%)	1 (0.1%)	4 (1.6%)	
Missing	2 (0.2%)	2 (0.3%)	0 (0.0%)	

HIV+				0.04^b^
Yes	20 (2.1%)	19 (2.6%)	1 (0.4%)	
No	947 (97.1%)	707 (96.6%)	240 (98.8%)	
Missing	8 (0.8%)	6 (0.8%)	2 (0.8%)	

Number of prenatal visits				0.54^a^ 0.08^c^
0	20 (2.1%)	11 (1.5%)	9 (3.7%)	
<8	895 (91.8%)	677 (92.5%)	218 (89.7%)	
8+	55 (5.6%)	39 (5.3%)	16 (6.6%)	
Missing	5 (0.5%)	5 (0.7%)	0 (0.0%)	
Median (IQR)	4 [3, 5]	4 [3, 5]	4 [3, 5]	

Antepartum, labor and delivery characteristics				
Onset of labor				<0.001^b^
Spontaneous	831 (85.2%)	638 (87.2%)	193 (79.4%)	
Augmented/Induced	121 (12.4%)	90 (12.3%)	31 (12.8%)	
Not applicable	22 (2.3%)	3 (0.4%)	19 (7.8%)	
Missing	1 (0.1%)	1 (0.1%)	0 (0.0%)	

Transferred during labor				<0.001^b^
No	496 (50.9%)	408 (55.8%)	88 (36.2%)	
Yes	478 (49.0%)	323 (44.1%)	155 (63.8%)	
Missing	1 (0.1%)	1 (0.1%)	0 (0.0%)	

Cervical exam on admission				0.56^c^ 0.46^a^
<4 cm (latent labor)	486 (49.8%)	365 (49.9%)	121 (49.8%)	
4+ cm (active labor)	460 (47.2%)	353 (48.2%)	107 (44.0%)	
Missing or “not done” or “not applicable”	29 (3.0%)	14 (1.9%)	15 (6.2%)	
Median (IQR)	3 [2, 7]	3 [2, 7]	3 [2, 7]	

Duration of labor				<0.001^c^
Not applicable	28 (2.9%)	6 (0.8%)	22 (9.1%)	
<12 hours	502 (51.5%)	413 (56.4%)	89 (36.6%)	
12–24hours	394 (40.4%)	290 (39.6%)	104 (42.8%)	
24+ hours	51 (5.2%)	23 (3.2%)	28 (11.5%)	
Missing	0 (0.0%)	0 (0.0%)	0 (0.0%)	

Antepartum hemorrhage				0.003^b^
No	953 (97.7%)	721 (98.5%)	232 (95.5%)	
Yes	21 (2.2%)	10 (1.4%)	11 (4.5%)	
Missing	1 (0.1%)	1 (0.1%)	0 (0.0%)	

Chorioamnionitis				0.64^b^
No	969 (99.4%)	728 (99.5%)	241 (99.2%)	
Yes	6 (0.6%)	4 (0.5%)	2 (0.8%)	
Missing	0 (0.0%)	0 (0.0%)	0 (0.0%)	

Antepartum preeclampsia/eclampsia/chronic hypertension				0.53^b^
No	929 (95.3%)	699 (95.5%)	230 (94.7%)	
Yes	45 (4.6%)	32 (4.4%)	13 (5.3%)	
Missing	1 (0.1%)	1 (0.1%)	0 (0.0%)	

Chorioamnionitis				0.64^b^
No	969 (99.4%)	728 (99.5%)	241 (99.2%)	
Yes	6 (0.6%)	4 (0.5%)	2 (0.8%)	
Missing	0 (0.0%)	0 (0.0%)	0 (0.0%)	

Antepartum preeclampsia/eclampsia/chronic hypertension				0.53^b^
No	929 (95.3%)	699 (95.5%)	230 (94.7%)	
Yes	45 (4.6%)	32 (4.4%)	13 (5.3%)	
Missing	1 (0.1%)	1 (0.1%)	0 (0.0%)	

Mode of delivery				<0.001^b^
Unassisted vaginal	708 (72.6%)	702 (95.9%)	6 (2.5%)	
Forceps-assisted	12 (1.2%)	6 (0.8%)	6 (2.5%)	
Vacuum-assisted	29 (3.0%)	21 (2.9%)	8 (3.3%)	
Cesarean	223 (22.9%)	1 (0.1%)	222 (91.3%)	
Missing	3 (0.3%)	2 (0.3%)	1 (0.4%)	

Gestational age at delivery				0.19^c^ 0.03^a^
Preterm	102 (10.5%)	82 (11.2%)	20 (8.2%)	
Term	870 (89.2%)	648 (88.5%)	222 (91.4%)	
Missing	3 (0.3%)	2 (0.3%)	1 (0.4%)	
Median (IQR)	39 [38, 40]	38 [38, 40]	39 [38, 40]	

Birthweight (grams)				0.50^c^ <0.001^a^
<2500	68 (7.0%)	54 (7.4%)	14 (5.8%)	
≥2500	858 (88.0%)	650 (88.8%)	208 (85.6%)	
Missing	49 (5.0%)	28 (3.8%)	21 (8.6%)	
Median (IQR)	3100 [2900, 3500]	3000 [2900, 3400]	3200 [3000, 3600]	

Multiple gestation				0.008^b^
Yes	928 (95.2%)	705 (96.3%)	223 (91.8%)	
No	47 (4.8%)	27 (3.7%)	47 (8.2%)	
Missing	0 (0.0%)	0 (0.0%)	0 (0.0%)	

Postpartum maternal complications^d^				
Postpartum blood transfusion				<0.001^b^
No	960 (98.5%)	729 (99.6%)	231 (95.1%)	
Yes	13 (1.3%)	3 (0.4%)	10 (4.1%)	
Missing	2 (0.2%)	0 (0.0%)	2 (0.8%)	

Postpartum antibiotics				<0.001^c^
No	884 (90.7%)	716 (97.8%)	168 (69.2%)	
Yes	86 (8.8%)	14 (1.9%)	72 (29.6%)	
Missing	5 (0.5%)	2 (0.3%)	3 (1.2%)	

Maternal length of hospitalization median (IQR)	1 [1, 2]	1 [1, 1]	3 [3, 4]	<0.001^a^
Missing	4 (0.0%)	3 (0.0%)	1 (0.0%)	

Postpartum neonatal complications^e^				
Five-minute apgar score median (IQR)	9 [8, 9]	9 [8, 9]	8 [7, 9]	<0.001^a^
Missing	47 (4.5%)	27 (3.0%)	20 (1.8%)	

Stillbirth				0.001^b^
Yes, fresh	29 (3.0%)	11 (1.5%)	18 (7.4%)	
Yes, macerated	12 (1.2%)	11 (1.5%)	1 (0.4%)	
No	886 (91.0%)	682 (93.3%)	204 (84.0%)	
Missing	48 (4.8%)	28 (3.7%)	20 (8.2%)	

Neonate status on day of discharge				<0.001^c^
Dead	55 (5.6%)	29 (4.0%)	26 (10.7%)	
Alive	915 (93.9%)	699 (95.5%)	216 (88.9%)	
Missing	5 (0.5%)	4 (0.5%)	1 (0.4%)	

Neonatal length of hospitalization, median (IQR)	1 [1, 3]	1 [1, 1]	3 [3, 4]	<0.001^a^
Missing	20 (2.0%)	13 (1.7%)	7 (3.0%)	

^a^Kruskall–Wallis test. ^b^Fisher's Exact test. ^c^Chi-squared test. ^d^No difference in postpartum hemorrhage, uterotonic use. ^e^No difference in bag-and-mask, intranasal oxygen, continuous positive airway pressure, intravenous fluid administration, neonatal antibiotic administration, and neonatal blood transfusion.

**Table 2:** (A) Multivariable model of characteristics associated with IESO attendant and (B) how outcomes are impacted by delivery by IESO as compared to midwife attendant.

Characteristic	RR	CI	*p*-value
(A) Multivariable poisson model with robust error variance of characteristics associated with IESO as attendanta			
Mode of delivery (unassisted vaginal birth reference)			
Forceps-assisted	88.4	28.7, 271.7	<0.001
Vacuum-assisted	45.2	14.3, 142.3	<0.001
Cesarean	161.8	59.9, 437.2	<0.001

(B) Individual logistic regressions, adjusted for significant findings in Table 4, to determine association of cesarean birth with outcomes significant in bivariate comparisons (Table 3)			
Maternal outcomes^b^			
Odds of postpartum hemorrhage	Model did not converge		
Odds of needing postpartum antibiotics	13.4	3.0, 60.2	0.001
Odds of longer hospitalization	Model did not converge		

Neonatal outcomes^b^			
Odds of having a higher apgar score	0.1	0.1, 0.7	0.02
Odds of live birth	0.2	0.1, 1.3	0.09
Odds of being alive at discharge from the hospital	0.1	0.1, 0.5	0.005
Odds of longer neonatal hospitalization	0.2	0.1, 2.1	0.18

^a^Variables included in the model without an association with the outcome: urban/rural residence, parity, history of cesarean birth, HIV status, onset of labor (spontaneous or not), duration of labor, antepartum hemorrhage, birthweight, and multiple gestation. ^b^Adjusted for mode of delivery.

**Table 3:** Sociodemographic and obstetric characteristics of women overall and by IESO versus physician attendant.

Characteristic	*N* (%) *N* = 263	GP/MD 20 (*n* = 7.6%)	IESO (*n* = 243, 92.4%)	*p* value
Sociodemographic and obstetric characteristics				
Age in years, median (IQR)	25 [21, 28]	25 [21.5, 26.5]	25 [21, 28]	0.98^a^
Missing	0 (0.0%)	0 (0.0%)	0 (0.0%)	0 (0.0%)

Education				0.55^b^
Unable to read & write	68 (25.9%)	4 (20.0%)	64 (26.3%)	
Read & write only	10 (3.8%)	2 (10.0%)	8 (3.3%)	
Primary school	105 (39.9%)	9 (45.0%)	96 (39.5%)	
Secondary school	35 (13.3%)	2 (10.0%)	33 (13.6%)	
Higher education	45 (17.1%)	3 (15.0%)	42 (17.3%)	
Missing	0 (0.0%)	0 (0.0%)	0 (0.0%)	

Religion				0.05^b^
Muslim	34 (12.9%)	0 (0.0%)	34 (14.0%)	
Orthodox christian	83 (31.6%)	4 (20.0%)	79 (32.5%)	
Catholic christian	0 (0.0%)	0 (0.0%)	0 (0.0%)	
Protestant	146 (55.5%)	16 (80.0%)	130 (53.5%)	
Jehovah's witness	0 (0.0%)	0 (0.0%)	0 (0.0%)	
Missing	0 (0.0%)	0 (0.0%)	0 (0.0%)	

Relationship status				1.0^b^
Single	5 (1.9%)	0 (0.0%)	5 (2.1%)	
Not single	257 (97.7%)	20 (100.0%)	237 (97.5%)	
Missing	1 (0.4%)	0 (0.0%)	1 (0.4%)	

Woreda				0.22^b^
Urban	175 (66.5%)	16 (80.0%)	159 (65.4%)	
Rural	88 (33.5%)	4 (20.0%)	84 (34.6%)	

Parity				0.11^b^
0	116 (44.1%)	5 (25.0%)	111 (45.7%)	
1	70 (26.6%)	8 (40.0%)	62 (25.5%)	
2	26 (9.9%)	4 (20.0%)	22 (9.0%)	
3+	51 (19.4%)	3 (15.0%)	48 (19.8%)	
Missing	0 (0.0%)	0 (0.0%)	0 (0.0%)	

Months since last delivery (parity 1+ *n* = 147)				0.27^b^ 0.10^a^
<24 months	10 (6.8%)	2 (13.3%)	8 (6.1%)	
24+ months	136 (92.5%)	13 (86.7%)	123 (93.2%)	
Missing	1 (0.7%)	0 (0.0%)	1 (0.7%)	
Median (IQR)	48 [36, 84]	48 [24, 60]	48 [36, 84]	

History of cesarean birth				0.14^b^
0	229 (95.4%)	15 (75.0%)	214 (88.1%)	
1	30 (3.9%)	5 (25.0%)	25 (10.3%)	
2+	4 (0.5%)	0 (0.0%)	4 (1.6%)	
Missing	0 (0.0%)	0 (0.0%)	0 (0.0%)	

HIV+				0.15^b^
Yes	2 (0.8%)	1 (5.0%)	1 (0.4%)	
No	259 (98.4%)	19 (95.0%)	240 (98.8%)	
Missing	2 (0.8%)	0 (0.0%)	2 (0.8%)	

Number of prenatal visits				0.08^c^ 0.88^a^
0	9 (2.1%)	0 (0.0%)	9 (3.7%)	
<8	238 (91.8%)	20 (100.0%)	218 (89.7%)	
8+	16 (5.6%)	0 (0.0%)	16 (6.6%)	
Missing	0 (0.0%)	0 (0.0%)	0 (0.0%)	
Median (IQR)	4 [3, 5]	4 [4, 4.5]	4 [3, 5]	

Antepartum, labor and delivery characteristics				
Onset of labor				0.05^b^
Spontaneous	206 (78.3%)	13 (65.0%)	193 (79.4%)	
Augmented/Induced	33 (12.6%)	2 (10.0%)	31 (12.8%)	
Not applicable	24 (9.1%)	5 (25.0%)	19 (7.8%)	
Missing	0 (0.0%)	0 (0.0%)	0 (0.0%)	

Transferred during labor				0.74^b^
No	96 (36.5%)	8 (40.0%)	88 (36.2%)	
Yes	167 (63.5%)	12 (60.0%)	155 (63.8%)	
Missing	0 (0.0%)	0 (0.0%)	0 (0.0%)	

Cervical exam on admission				0.48^c^ 0.82^a^
<4 cm (latent labor)	129 (49.1%)	8 (40.0%)	121 (49.8%)	
4+ cm (active labor)	117 (44.5%)	10 (50.0%)	107 (44.0%)	
Missing or “not done” or “not applicable”	17 (6.5%)	2 (10.0%)	15 (6.2%)	
Median (IQR)	3 [2, 7]	4 [2, 8]	3 [2, 7]	

Duration of labor				0.19^b^
Not applicable	26 (9.9%)	4 (20.0%)	22 (9.1%)	
< 12 hours	97 (36.9%)	8 (40.0%)	89 (36.6%)	
12–24 hours	112 (42.6%)	8 (40.0%)	104 (42.8%)	
24+ hours	28 (10.6%)	0 (0.0%)	28 (11.5%)	
Missing	0 (0.0%)	0 (0.0%)	0 (0.0%)	

Antepartum hemorrhage				0.003^b^
No	953 (97.7%)	721 (98.5%)	232 (95.5%)	
Yes	21 (2.2%)	10 (1.4%)	11 (4.5%)	
Missing	1 (0.1%)	1 (0.1%)	0 (0.0%)	

Chorioamnionitis				0.64^b^
No	969 (99.4%)	728 (99.5%)	241 (99.2%)	
Yes	6 (0.6%)	4 (0.5%)	2 (0.8%)	
Missing	0 (0.0%)	0 (0.0%)	0 (0.0%)	

Antepartum preeclampsia/eclampsia/chronic hypertension				1.0^b^
No	251 (95.4%)	19 (95.0%)	232 (95.5%)	
Yes	12 (4.6%)	1 (5.0%)	11 (4.5%)	
Missing	0 (0.0%)	0 (0.0%)	0 (0.0%)	

Mode of delivery				<0.001^b^
Unassisted vaginal	14 (5.3%)	8 (40.0%)	6 (2.5%)	
Forceps-assisted	8 (3.0%)	2 (10.0%)	6 (2.5%)	
Vacuum-assisted	8 (3.0%)	0 (0.0%)	8 (3.3%)	
Cesarean	232 (88.2%)	10 (50.0%)	222 (91.3%)	
Missing	1 (0.4%)	0 (0.0%)	1 (0.4%)	

Gestational age at delivery				0.68^b^ 0.03^a^
Preterm	22 (8.4%)	2 (10.0%)	20 (8.2%)	
Term	240 (91.2%)	18 (90.0%)	222 (91.4%)	
Missing	1 (0.4%)	0 (0.0%)	1 (0.4%)	
Median (IQR)	39 [38, 40]	38 [38, 40]	39 [38, 40]	

Birthweight (grams)				0.14^b^ 0.57^a^
<2500	17 (6.4%)	3 (15.0%)	14 (5.8%)	
≥2500	224 (85.2%)	16 (80.0%)	208 (85.6%)	
Missing	22 (8.4%)	1 (5.0%)	21 (8.6%)	
Median (IQR)	3200 [3000, 3600]	3200 [2700, 3500]	3200 [3000, 3600]	

Multiple gestation				1.0^b^
Yes	242 (95.2%)	19 (95.0%)	223 (91.8%)	
No	21 (4.8%)	1 (5.0%)	20 (8.2%)	
Missing	0 (0.0%)	0 (0.0%)	0 (0.0%)	

Postpartum maternal complications^d^				
Maternal length of hospitalization median (IQR)	3 [3, 4]	3 [1, 4]	3 [3, 4]	0.02^a^
Missing	1 (0.X%)	0 (0.0%)	1 (0.X%)	

Postpartum neonatal complications^e^				
Neonatal length of hospitalization, median (IQR)	3 [3, 4]	2 [1, 3.5]	3 [3, 4]	0.01^a^
Missing	8 (x.0%)	0 (0.0%)	8 (x.0%)	

^a^Kruskall–Wallis test. ^b^Fisher's Exact test. ^c^Chi-squared test. ^d^No difference in postpartum hemorrhage, uterotonic use, maternal postpartum blood transfusion, and maternal postpartum antibiotics. ^e^No difference in bag-and-mask, intranasal oxygen, continuous positive airway pressure, intravenous fluid administration, neonatal antibiotic administration, neonatal blood transfusion, neonate status on day of discharge, neonatal status at birth, and neonatal Apgar score.

**Table 4:** (A) Multivariable model of characteristics associated with IESO attendant and (B) how outcomes are impacted by IESO delivery.

Characteristic	RR	CI	*p* value
(A) Poisson regression of characteristics associated with IESO as attendant as compared to GP or Ob/Gyn resident or attending			
Religion (compared to muslim)	0.9	0.9, 0.9	0.01
Catholic christian			

Labor onset (compared to “not applicable”			
Spontaneous	1.3	1.1, 1.6	0.03
Augmented/Induced	1.2	1.0, 1.5	0.07
Risk of antepartum hemorrhage	1.1	0.1, 2.0	0.2

Mode of delivery (unassisted vaginal birth reference)			
Forceps-assisted	1.8	0.9, 3.7	0.11
Vacuum-assisted	2.4	1.3, 4.4	0.003
Cesarean	2.3	1.3, 4.2	0.006

(B) Individual logistic regressions, adjusted for significant findings in Table 4, to determine association of cesarean birth with outcomes significant in bivariate comparisons (Table 3)^a^			
Maternal outcomes			
Odds of longer hospitalization	Model did not converge		
Neonatal outcomes			
Odds of longer neonatal hospitalization	1.2	0.1, 9.8	0.87

^a^Adjusted for religion, labor onset, and mode of delivery.

## Data Availability

Data are available upon request.
